# Cross-talk between signalling pathways and the multidrug resistant protein MDR-1

**DOI:** 10.1054/bjoc.2001.2044

**Published:** 2001-10

**Authors:** S Ding, M Chamberlain, A McLaren, L-b Goh, I Duncan, C R Wolf

**Affiliations:** Biomedical Research Centre, ICRF Molecular Pharmacology Unit, Department of Obstetrics and Gynaecology, Ninewells Hospital and Medical School, Dundee, DD1 9SY, UK

**Keywords:** MAP kinase, MDR-1, drug resistance, ovarian cancer, Taxol

## Abstract

The multidrug resistant protein MDR-1 has been associated with the resistance to a wide range of anti-cancer drugs. Taxol is a substrate for this transporter system and is used in the treatment of a wide range of human malignancies including lung, breast and ovarian cancer. We have generated a series of ovarian cell lines resistant to this compound, all of which overexpress MDR-1 through gene amplification. We present novel evidence that a constitutive activation of the ERK1/2 MAP kinase pathway was also observed although the level of active JNK and p38 remained unchanged. Inhibition of the ERK1/2 MAP kinase pathway using UO126 or PD098059 re-sensitised the Taxol resistant cells at least 20-fold. Importantly, when Mdr-1 cDNA was stably expressed in the wild-type cell line to generate a highly Taxol-resistant sub-line, 1847/MDR5, ERK1/2 MAP kinases again became activated. This result demonstrated that the increased activity of the signalling pathway in the Taxol-resistant lines was directly attributable to MDR-1 overexpression and was not due to the effects of Taxol itself. Additionally, we demonstrated that inhibition of the P13K pathway with LY294002 sensitised the MDR-1-expressing 1847/TX0.5 cells and 1847/MDR5 cells at least 10-fold but had no effect in the wild-type cells. This finding suggests a possible role for this pathway, also, in the generation of resistance to Taxol. © 2001 Cancer Research Campaign  http://www.bjcancer.com


*British Journal of Cancer* (2001) **85**(8), 1175–1184
© 2001 Cancer Research Campaign
doi: 10.1054/ bjoc.2001.2044, available online at http://www.idealibrary.com on IDEAL
http://www.bjcancer.com

### Cross-talk between signalling pathways and the multidrug resistant protein MDR-1

**S Ding[1], M Chamberlain[1,2], A McLaren[1,2], L-b Goh[1], I Duncan[3] and CR Wolf[1,2]**

[1]Biomedical Research Centre, [2]ICRF Molecular Pharmacology Unit, [3]Department of Obstetrics and Gynaecology, Ninewells Hospital and Medical School, Dundee, DD1 9SY, UK

**Summary** The multidrug resistant protein MDR-1 has been associated with the resistance to a wide range of anti-cancer drugs. Taxol is a substrate for this transporter system and is used in the treatment of a wide range of human malignancies including lung, breast and ovarian cancer. We have generated a series of ovarian cell lines resistant to this compound, all of which overexpress MDR-1 through gene amplification. We present novel evidence that a constitutive activation of the ERK1/2 MAP kinase pathway was also observed although the level of active JNK and p38 remained unchanged. Inhibition of the ERK1/2 MAP kinase pathway using UO126 or PD098059 re-sensitised the Taxol resistant cells at least 20-fold. Importantly, when Mdr-1 cDNA was stably expressed in the wild-type cell line to generate a highly Taxol-resistant sub-line, 1847/MDR5, ERK1/2 MAP kinases again became activated. This result demonstrated that the increased activity of the signalling pathway in the Taxol-resistant lines was directly attributable to MDR-1 overexpression and was not due to the effects of Taxol itself. Additionally, we demonstrated that inhibition of the P13K pathway with LY294002 sensitised the MDR-1-expressing 1847/TX0.5 cells and 1847/MDR5 cells at least 10-fold but had no effect in the wild-type cells. This finding suggests a possible role for this pathway, also, in the generation of resistance to Taxol. © 2001 Cancer Research Campaign http://www.bjcancer.com

**Keywords**: MAP kinase; MDR-1; drug resistance; ovarian cancer; Taxol

Chemotherapy often results in the emergence of multidrug resistance in cancer cells and over-expression of P-glycoprotein (MDR-1) is widely accepted to be a contributor to the resistance to some chemotherapeutic reagents. Reversal of multidrug resistance, therefore, is important for the successful treatment of many tumours following drug treatment. However, clinical trials utilising inhibitors of MDR-1 have not been that successful, indicating that unknown mechanisms may also contribute to MDR-1-mediated resistance.

Mammalian cells possess the intrinsic capacity to initiate cell suicide through apoptosis. Three types of stimuli normally initiate this process: the deprivation of survival factors, signals through pro-apoptotic receptors or cell-damaging stress (Kinloch et al, 1999). In recent years, it has been demonstrated that chemical toxicity is accompanied by the induction of a large number of stress or adaptive response genes (Keyse, 1995). External or internal stimuli trigger a cascade of intracellular signals leading to proliferation, differentiation or apoptosis. Some tumour cells may actually avoid the ‘apoptotic safety-net’ because the damage signal generated by the antitumour agent fails to activate the necessary pathways required to initiate apoptosis. Indeed, activation of the signalling pathways regulating cell survival has been shown to play an anti-apoptotic role after treatment with various chemotherapeutic drugs, ionising radiation or oxidant injury (Dent et al, 1998; Wang et al, 1998; Plo et al, 1999).

*Received 12 March 2001*
*Revised 3 July 2001*
*Accepted 16 July 2001*

*Correspondence to:* CR Wolf

Many signal-transduction pathways have now been described and well characterised. Of these, the mitogen-activated protein kinase (MAPK) family has emerged as one of the most important membrane-to-nucleus signalling mechanisms, functioning as a mediator of cellular responses to a variety of cellular stimuli (Hunter, 1997). The MAPK family has been classified into 3 distinct subfamilies: the extracellular signal-regulated protein kinases (ERKs) including ERK1 and ERK2, the stress-activated c-Jun N-terminal protein kinase (JNKs) and p38 kinase. ERKs are activated by growth factors, cytokines and hormones. Constitutive activation of this pathway, for example by mutations in the Ras oncogene, is sufficient to promote cell transformation (Mansour et al, 1994; Hoshino et al, 1999). In addition, exposure of cells to antitumour agents such as Taxol can activate the ERK1/2 MAP kinase (Schulte et al, 1996; Blagosklonny et al, 1999). Cisplatin is also known to activate the JNK pathway in human ovarian cancer cells (Hayakawa et al, 1999). Inhibitors of the ERK1/2 MAP kinase cascade such as PD184352 have been shown to inhibit the growth of human colon cancer xenografts in mice by as much as 80% (Sebolt-Leopold et al, 1999). Furthermore, studies have also shown that the activation of the ERK1/2 MAP kinase pathway plays a critical role in leukaemia cell survival after treatment with chemotherapeutic drugs and ionising radiation (Dent et al, 1998).

In addition to the ERK1/2 MAP kinase cascade, phosphatidylinositol-3-kinase (PI3K) is also an effector of Ras signalling (Franke et al, 1997; Kauffmann-Zeh et al, 1997). It is assumed that 3′-phosphoinositides generated by P13K act as second messengers and can bind to the pleckstrin homology (PH) domain of Akt/PKB, resulting in a conformational change of the protein. This promotes Akt/PKB activation through the phosphorylation at residues Thr-308 and Ser-473 by upstream kinases. Akt/PKB has emerged as a putative downstream effector that suppresses apoptosis (Leevers et al, 1999). It has recently been shown that approximately 40% of ovarian cancers contain increased copy number of the PI3KCA gene and increased PI3K activity (Shayesteh et al, 1999). The consequent activation of Akt/PKB is considered to be an important factor in the progression of ovarian cancer (Yuan et al, 2000). Recently it has been found that, in certain circumstances, PI3K plays a role in receptor-driven activation of the ERK1/2 MAP kinase signalling cascade (Wennstrom and Downward, 1999; Scheid and Woodgett, 2000). Although the 2 cascades were once thought to be distinct and act in parallel, induction of PI3K may actually deliver a co-operative activation signal to the ERK 1/2 MAP kinase pathway. Cross-talk between the 2 cascades may play a crucial role in the regulation of cell growth and survival.

The chemotherapeutic agent, Taxol, has been used in the treatment of a wide range of human malignancies including lung, head and neck, breast and ovarian cancer (Ling et al, 1998). The short-term survival rate of these cancers has improved. However, the 5-year survival rate has remained essentially unchanged. The major limitation to successful treatment is that the initial round of treatment with Taxol does not kill all the tumour cells and invariably the tumour returns in a manner that is resistant to further drug treatment.

On the basis of these observations we were interested in determining whether these signalling pathways play a role in chemically induced cell death, and whether they are involved in tumour cell chemoresistance. To this end we investigated the role of these pathways in the resistance of ovarian cancer cells to the important anti-tumour agent Taxol.

### MATERIALS AND METHODS

### Cell lines and cell culture

Human ovarian cancer cell line A1847 (Eva et al, 1982) was obtained from the Imperial Cancer Research Fund (Clare Hall, London). Cells were grown as monolayers in RPMI medium supplemented with 10% fetal calf serum, (100 U ml$^{-1}$) penicillin and (100 µg ml$^{-1}$) streptomycin (GIBCOBRL, Paisley, UK) at 37˚C in a 5% $CO_2$ atmosphere. A1847 cells were exposed to 200 ng ml$^{-1}$ EMS (ethyl methane sulfonate) for 24 hours, allowing the cells to recover for 48 hours, then treating with 1 nM Taxol. These cells were allowed to adapt to this concentration of Taxol for 3 passages before the Taxol concentration was increased to 2.5 nM. This incremental exposure to Taxol was continued until cells had become resistant to 3 µM Taxol. After each incremental change in the Taxol concentration, individual colonies were isolated with cloning cylinders to generate cell lines which would grow in the presence of 0.5 µM (A1847/TX0.5), 1 µM (A1847/TX1) and 3 µM (A1847/TX3) Taxol, respectively.

### Plasmid construction and establishment of the cell lines expressing MDR-1 by DNA transfection

Full-length human Mdr-1 cDNA (4.2 kb) was excised from the plasmid pBluescript/Mdr-1 (Kioka et al, 1989) by *BstU*I/*Xho*I digestion and ligated into the *Eco*RV/*Xho*I sites of an expression vector pcDNA3 (Invitrogen) containing a G418 gene to construct the plasmid pcDNA/Mdr-1. DNA transfection was carried out as described previously (Ding et al, 1997). After transfection with expression plasmid pcDNA/Mdr-1, cells were selected with G418 (GIBCOBRL, 800 µg ml$^{-1}$). Individual colonies were isolated with cloning cylinders to generate cell lines and cell lines A1847/MDR5 and A1847/MDR3 were used in further study. The control line (A1847/pcDNA) was generated from cells transfected with the vector alone and selected using G418. After isolation of resistant clones, the concentration of G418 was changed to 400 µg ml$^{-1}$.

### Western blot analysis

Cells were harvested by cell lifter and lysed in 0.1 ml lysis buffer containing 50 mM Tris-HCl (pH 7.6), NaCl (100 mM), EDTA (2 mM), 1% (v/v) NP40, sodium orthovanadate (1 mM) and phenylmethylsulfonyl fluoride (1 mM). Protein samples were sonicated using an MSE Soniprep (two 5 second bursts at amplitude microns of 12 with sample kept on ice). Proteins samples were separated by sodium dodecyl sulfate polyacrylamide gel electrophoresis (SDS/PAGE), transferred to a Nitrocellulose membrane and probed with primary antibody as detailed previously (Ding et al, 1997). Immunoblots were developed using the enhanced chemiluminescence (ECL Plus) Western blotting kit (Amersham).

### MTT assay for cytotoxicity

Taxol-induced cytotoxicity was determined using the MTT assay (Mosmann, 1983). Briefly, cells were trypsinised and plated out at a density of 3000 cells per well into 96 well plates. Cells were cultured overnight and re-fed with fresh medium with various concentrations of Taxol ($1.0 \times 10^{-9}$–$2.0 \times 10^{-5}$ M). For the pre-treatment, cells were treated with PD098059 (30 µM) or UO126 (20 µM) for 2 hours. Then cells were treated with various concentrations of Taxol plus a fixed concentration of inhibitors. 72 h later 50 µl of 2 mg ml$^{-1}$ MTT (3-(4,4-dimethylthiazol-2-yl)-2,5-diphenyltetrazolium bromide) (Sigma, Poole, UK) in PBS were added to each well, incubated for 4 h at 37˚C and the formazan crystals formed were dissolved in 50 µl of DMSO. The optical density was recorded at 570 nm on a microplate reader (BIO-RAD). Cytotoxicity is expressed as $IC_{50}$ for each of the cell lines, this is the concentration of drug that caused a 50% reduction in the absorbance at 570 nm relative to untreated cells (control) or cells treated with inhibitor alone.

### Phosphorylation of MDR-1

Cells were plated into 6-well culture plates and cultured in RPMI medium supplemented with 10% fetal calf serum, (100 U ml$^{-1}$) penicillin and (100 µg ml$^{-1}$) streptomycin (GIBCOBRL, Paisley, UK) at 37˚C in a 5% $CO_2$ atmosphere overnight. The growth medium was replaced with $PO_4$-free medium for 1 h, and then supplemented with 250 µCi of $^{32}PO_4$ per well. After 2 h incubation at 37˚C, the culture medium was replaced with unlabelled medium. Cells were treated with PD098059 (30 µM) or staurosporine (50 nM) for 1 h. Reactions were terminated by lysing the cells in ice-cold lysis buffer. $^{32}PO_4$-labelled MDR-1 was immunoprecipitated by the incubation with anti-Pgp antibody C219 or an anti-peptide antibody. The results shown are representative of at least 2 separate observations using either method.

### Transporter assays

Cells ($5 \times 10^5$) were seeded into 6-well culture plates and cultured overnight. The growth medium was replaced with fresh

 *© 2001 Cancer Research Campaign*

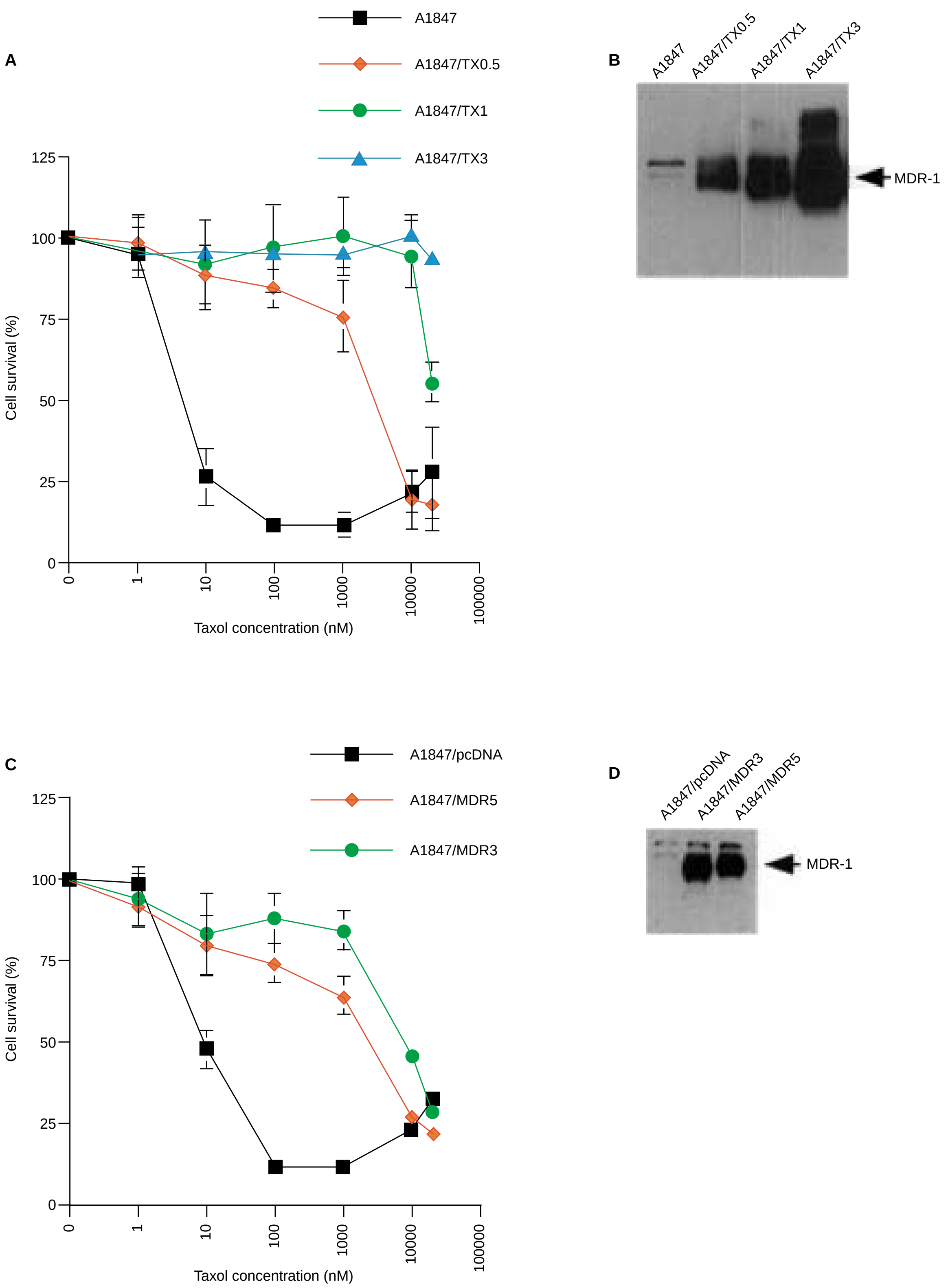

**Figure 1** Correlation between the degree of Taxol resistance and expression of MDR-1 in parental and Taxol-resistant derivatives of A1847 cells. (**A**) Cytotoxicity of Taxol in parental A1847 cells and in sub-lines growing in the presence of 0.5, 1.0 and 3.0 $\mu$M Taxol respectively. Cell survival was determined following a 72 hour incubation in the presence of various concentrations of Taxol from $1.0 \times 10^{-9}$ to $2.0 \times 10^{-5}$ M using the MTT assay as described in the Methods. The percentage of viable cells was calculated by dividing the MTT activity of treated cells by those treated with DMSO alone. The results shown are mean ± SD of at least triplicate wells from 3 independent experiments. (**B**) Expression of MDR-1 protein in A1847 cells and in Taxol-resistant sublines. MDR-1 expression was analysed by Western blot analysis using 25 $\mu$g of total cellular protein per lane. Proteins were separated on 7.5 % SDS-PAGE gels, transferred to a nitrocellulose membrane and probed with anti-MDR-1 monoclonal antibody (1/200 dilution) as described in the Methods. (**C**, **D**) Cytotoxicity of Taxol and expression of MDR-1 in transfected cell lines (MDR3 and MDR5) and in control cell line A1847/pcDNA transfected with vector alone. Assays were carried out as described in **A** and **B**

© 2001 Cancer Research Campaign 

medium. Cells were pre-treated with DMSO (as control), PD098059(30 μM) for 2 h, or with verapmil (25 μM) for 30 min, and then supplemented with 1 μM vinblastine (the ratio of labelled to unlabelled vinblastine was 100:1, and the specific activity of $^3$H-vinblastine was 12.7 Ci mmol$^{-1}$) for 20 min. Reactions were terminated by aspirating the radioactive medium, and cells were washed with PBS 3 times. Cells were lysed in 0.5 ml of 0.4 M NaOH, and then neutralised with 0.5 ml of 0.4 M ammonium acetate, pH 6.6. Radioactivity was determined in duplicate in a liquid scintillation counter WALLAC 1409. Cell number in control wells was counted and results were then normalised against cell number (1 × 10$^6$).

### RESULTS

### Over-expression of MDR-1 in Taxol-resistant sublines of A1847

We generated a panel of resistant sub-cell lines of the ovarian cancer cell line A1847. Cells were exposed to a mutagen EMS (ethyl methane sulfonate) for 24 h and then selected in increasing concentrations of Taxol (from 2.5 nM to 10 μM). Single colonies were isolated to generate clonal cell lines which would grow in the presence of, 0.5 μM (A1847/TX0.5), 1 μM (A1847/TX1) and 3 μM (A1847/TX3) Taxol respectively. These cell lines exhibited very marked increases in Taxol resistance in cell cytotoxicity assays (Figure 1A). The degree of the resistance of each line correlated with the final Taxol concentration to which it had originally been exposed. The $IC_{50}$ value for cell line A1847/TX0.5 (8.8 ± 2.9 μM) was more than 1000-fold higher than that of the parental cell line (7.1 ± 2.8 nM). In the case of A1847/TX3, the $IC_{50}$ could not be determined because even at the solubility limit of Taxol (100 μM) more than 70% of the cells remained viable.

The multidrug resistance transporter MDR-1 has been associated with resistance to Taxol (Sandor et al, 1998). We therefore determined whether the expression of this gene as well as certain other genes implicated in drug resistance such as glutathione S-transferase pi (GSTP1) had changed. No change in GSTP1 levels were seen (data not shown). However, a marked elevation in MDR-1 protein was observed in the resistant cells as shown (Figure 1B) which correlated well with the degree of resistance. Furthermore, Southern blot analysis revealed that the overexpression was due to amplification of the Mdr-1 gene (data not shown). To determine whether the increased expression of MDR-1 was responsible for the Taxol-resistance, wild-type A1847 cells were stably transfected with the Mdr-1 cDNA to generate the cell lines A1847/MDR5 and A1847/MDR3 (Figure 1D). These cells exhibited 300-fold (A1847/MDR5) and 850-fold (A1847/MDR3) higher Taxol resistance than cells transfected with vector alone (A1847/pcDNA) (Figure 1C). This resistance correlated well with the levels of MDR-1 determined by Western blot analysis. Although this finding together with other published results (Bosch and Groop, 1996; Sandor et al, 1998) shows the role of MDR-1 in drug resistance, it was reported recently that Taxol resistance can develop via either MDR-1-mediated or non-MDR-1-mediated mechanisms in ovarian and other cancers (Sarris et al, 1996; Parekh et al, 1997). Therefore, other genes such as those involved with signal transduction pathways may be involved.

### Role of cell signalling

Cell signalling cascades are important in both tumour pathogenesis and progression. Antitumour agents are known to activate the MAP kinase pathways and as a consequence affect tumour cell drug resistance (Dent et al, 1998). We therefore studied their expression and activity in the A1847 cell line and its drug resistant sub-lines.

Exposure of the parental A1847 cells to Taxol resulted in a transient activation of ERK1/2, the activation occurring 30 min after treatment with Taxol, and reaching a maximum at 8 h, declining thereafter (Figure 2A). In order to characterise the role of these signalling pathways in Taxol-resistant cell lines immunoblots on the cell lines were carried out with antibodies against phosphorylated ERK1/2. Interestingly, constitutive activation of ERK1/2 was

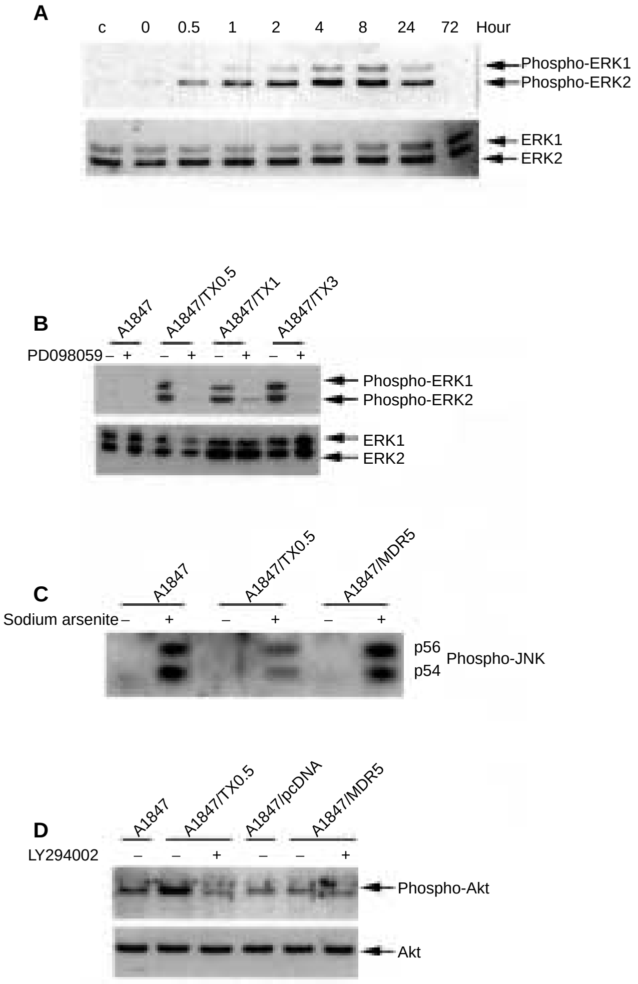

**Figure 2** Changes in cell signalling pathways in Taxol resistant cells. Total cell lysates were prepared from cells treated with DMSO alone (–) or cells treated with specific inhibitors or activators of the pathways being investigated (+). Protein samples (25 μg) were run on 10% SDS-PAGE gels and probed with antibodies either specific to the phosphorylated protein (top panels) or to the protein in general (**A**, **B** and **D**) allowing both the level of the protein and its activation state to be determined. (**A**) Activation of the ERK 1/2 MAP kinase pathway in A1847 cells following exposure to Taxol (1 μM). (**B**) Constitutive activation of ERK1/2 MAP kinase in the Taxol-resistant cell lines. (+) represents cells treated with PD098059 (30 μM). (**C**) JNK activity in the A1847 cells. The presence of inducible JNK was tested by incubation in the presence of 0.5 mM sodium arsenite (+). (**D**) Activity of Akt/PKB in untreated and treated cells with LY294002 (20 μM)

*© 2001 Cancer Research Campaign*

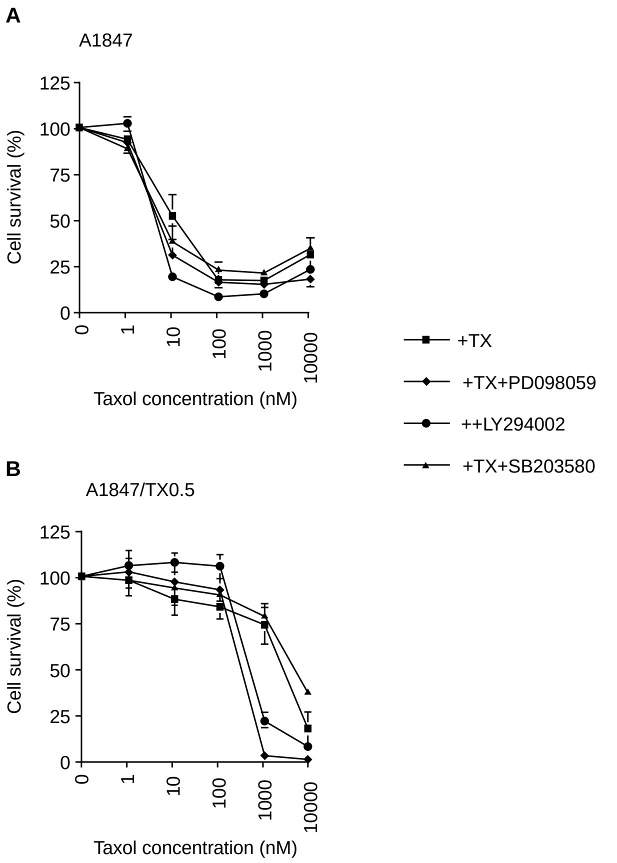

**Figure 3** Effect of signalling inhibitors on Taxol toxicity in wild type A1847 and A1847/TX0.5 cells. MTT cytotoxicity assays were carried out as described in Figure 1A. Cells were pre-treated with PD098059 (30 μM for 2 hours), LY294002 (20 μM for one hour), SB203580 (10 μM for one hour) respectively. Cells were then continuously exposed to Taxol plus the same concentration of inhibitors for 3 days before assay for cell survival. The percentage of viable cells was calculated by dividing the absorbance of cells treated with inhibitor alone by those treated with Taxol plus inhibitor. The results shown are mean ± SD of at least triplicate wells from 3 independent experiments observed in all the Taxol-resistant cells in the absence of Taxol, but not in the parental cells. This was completely abrogated by the MEK1 inhibitor PD098059 (Alessi et al, 1995) (Figure 2B). The expression and activation of JNK or p38 was also determined. No constitutive activity was observed in any of the cell lines (Figure 2C), however an inducible JNK activity was shown to be present in all cell lines following exposure to the known activator of this pathway, sodium arsenite.

To determine whether the P13K pathway was activated in A1847 or A1847/TX0.5 cells, immunoblot analysis using an antibody against phospho-Akt/PKB was carried out. This showed that there was a low level of activity in wild-type cells and the level of activation was increased in the A1847/TX0.5 cells (Figure 2D).

In order to further characterise the role of these pathways in the generation of drug resistance, several small molecule chemical inhibitors were tested to determine their effect on the Taxol-resistant phenotype. In view of the constitutive activation of ERK1/2 MAP kinase, we determined whether PD098059 could increase the Taxol sensitivity. PD098059 sensitised both wild-type (2-fold, $P < 0.05$) and Taxol-resistant A1847TX0.5 cells (26-fold) (Figure 3A and Table 1). In addition, LY294002, an inhibitor of the PI3K pathway (Davies et al, 2000) increased the Taxol sensitivity in the Taxol-resistant A1847/TX0.5 cells (20-fold) and 1847/MDR5 cells (10-fold), but had no effect on the wild-type cells (Table 1). Treatment with LY294002 blocked the phosphorylation of Akt/PKB in the cells. The inhibitor of the p38 pathway, SB203580 (Davies et al, 2000) did not affect the Taxol sensitivity in any of the cell lines tested (Figure 3). The above data suggest that both the ERK1/2 and PI3K, but not the p38 pathway may play a role in the generation of Taxol resistance in A1847 cells.

### Evaluation of whether the constitutive activation of ERK1/2 and PI3K are linked to the over-expression of MDR-1

The transient activation of ERK1/2 following Taxol exposure has been associated with Taxol treatment (Schulte et al, 1996; Blagosklonny et al, 1999). Therefore, the constitutively elevated activity in the Taxol-resistant cell lines could be a consequence of the selection process. There is a report on the constitutive activation of ERK1/2 in Taxol-resistant breast tumour cells, which was attributed to the presence of residual Taxol in these MDR-1-expressing cells (Emanuel et al, 1999). In addition, the elevated PI3K activity could also contribute to such a selective advantage. The alternative possibility is that these pathways are metabolically linked with MDR-1 over-expression. In order to evaluate these possibilities we measured ERK1/2 and PI3K activities in the cell lines transfected with Mdr-1 (Figure 4A). Intriguingly, in these cells, which were not exposed to Taxol, a constitutive activation of ERK1/2 was also observed. The degree of activation correlated with the level of MDR-1 expression (Figures 4A and 1D). No activation in the control cell line A1847/pcDNA was observed. However, PKB was not activated by MDR-1 overexpression in the

**Table 1** Effects of signalling inhibitors on Taxol sensitivity

| Cell line | Taxol | Taxol+PD098059 | Taxol+UO126 | Taxol+LY294002 |
|---|---|---|---|---|
| A1847 | 7.1 ± 2.8 nM | 3.5 ± 0.8 nM | ND | [†]6.2 ± 0.1 nM |
| A1847/TX0.5 | 8.80 ± 2.90 μM | 0.34 ± 0.02 μM | ND | 0.46 ± 0.01 μM |
| A1847/pcDNA | 5.8 ± 0.4 nM | 2.8 ± 0.1 nM | 2.0 ± 0.5 nM | [†]5.8 ± 0.5 nM |
| A1847/Mdr5 | 2.12 ± 0.61 μM | 0.02 ± 0.01 μM | 0.10 ± 0.01 μM | 0.22 ± 0.01 μM |

The values shown are $IC_{50}$ values obtained from MTT assays using the cell lines: A1847 wild type; /TX0.5 Taxol resistant; /pcDNA3 transfected with vector alone; /Mdr5 transfected with Mdr-1 cDNA and expressing Mdr-1. $IC_{50}$ values were obtained in taxol-treated cells or cells treated with taxol plus: PD098059 (30 μM), UO126 (20 μM) or LY294002 (20 μM). Values are mean ± SD from a minimum of three experiments. [†], All data when compared to appropriate control is significant (at least $P < 0.5$) with the exception of ([†]) where $P > 0.5$, and not significant.

© 2001 Cancer Research Campaign 

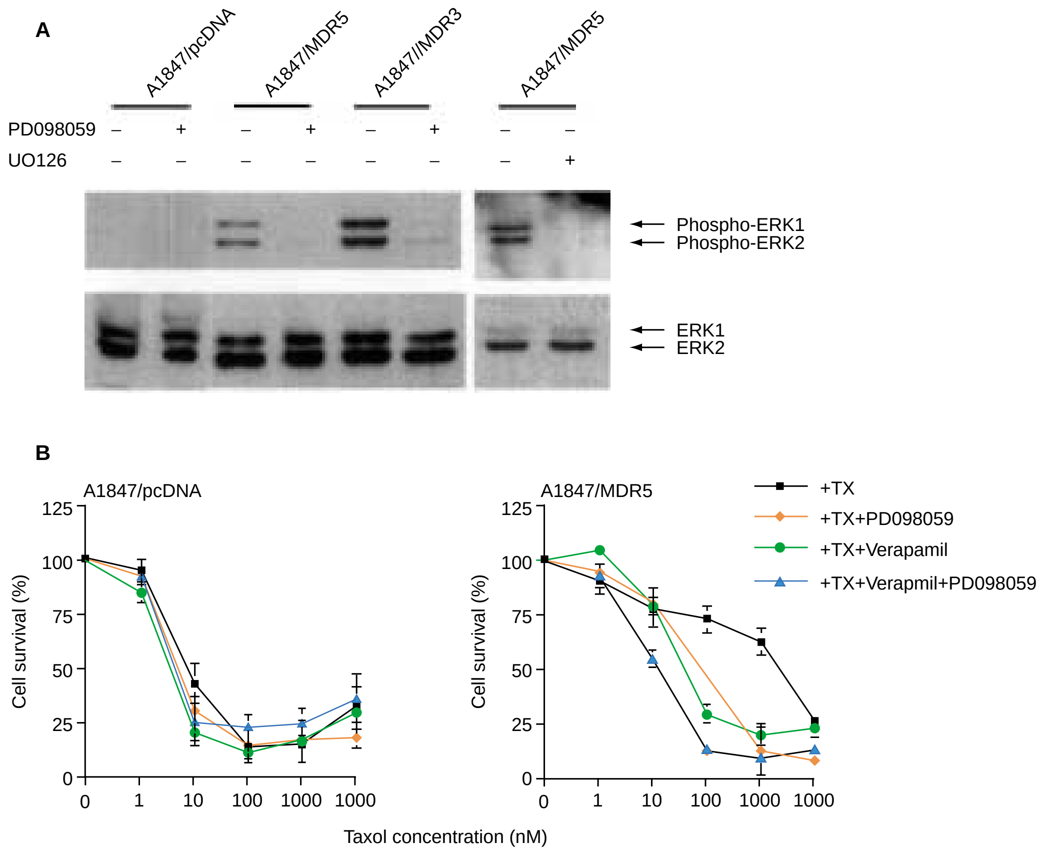

**Figure 4** Constitutive ERK 1/2 activity in MDR-1 transfected A1847 cells and the effect of inhibition of this pathway on Taxol cytotoxicity. (**A**) Phospho-ERK1/2 and the overall ERK1/2 levels were determined in the control cell lines (A1847/pcDNA) or cell lines stably transfected and expressing MDR-1 (MDR3 and MDR5). The analysis was carried out and probed as described in Figure 2 and 'Materials and Methods'. (**B**) Effect of ERK 1/2 MAP kinase inhibition on Taxol cytotoxicity. MTT cytotoxicity assays were carried as described in Figure 3

A1847/MDR5 cells (Figure 2D), indicating that the elevated PKB activity observed in A1847/TX0.5 cells is unrelated to expression of MDR-1.

### Reversal of Taxol cytotoxicity in MDR-1 transfectants

We then investigated whether the inhibition of ERK1/2 MAP kinase pathway would also affect the resistant phenotype of the Mdr-1 transfected cells. When A1847/MDR5 cells were treated with PD098059 (30 µM) an 88-fold sensitisation to Taxol cytotoxicity was observed (Figure 4B, Table 1). This change was paralleled by a complete inhibition of ERK1/2 activity (Figure 4A). The effects of PD098059 treatment were similar to the potent inhibitor of MDR-1 protein, verapamil (Tsuruo et al, 1981). Verapamil is a classical inhibitor of MDR-1 and therefore a positive control in these experiments. To assess whether PD098059 and verapamil could synergise and enhance the cytotoxicity of Taxol the 2 compounds were applied together. This is not an unreasonable hypothesis if PD098059 and verapamil affect MDR-1 function by different mechanisms. PD098059 in combination with verapamil further sensitised the cells to Taxol (2-fold, $P < 0.05$). UO126 (20 µM), an alternative inhibitor of the ERK 1/2 MAP kinase pathway (Favata et al, 1998), also sensitised the A1847/MDR5 cells to Taxol (20-fold) (Table 1) and completely blocked the phosphorylation of ERK1/2 (Figure 4A), supporting the role of this pathway in Taxol resistance. However, the fact that U0126 was less efficacious perhaps suggests an additional target for PD098059? Interestingly, treatment of the A1847/MDR5 cells with the PI3K inhibitor LY294002 increased Taxol sensitivity some 10-fold (Table 1), although the level of phospho-Akt remained unchanged in the cells compared to A1847/pcDNA cells (Figure 2C).

### Interrelationship between ERK1/2 MAP kinase and MDR-1

### *Phosphorylation*

It is known that MDR-1 is phosphorylated on serine residues 669 and 681 in vivo by the protein kinases PKA and PKC. The evidence, to date, that phosphorylation of MDR-1 is directly involved in the regulation of drug transport activity is, however, contradictory since alterations in the phosphorylation state have been associated with both enhancement and inhibition of drug transport (Smith and Zilfou, 1995). Treatment with the protein kinase inhibitor staurosporine decreases phosphorylation of MDR-1 and increases drug accumulation (Castro et al, 1999). However, we found that treatment with this compound had no effects on the response of A1847/TX0.5 and A1847/MDR5 cells to Taxol (data not shown). To further explore the possible role of constitutively activated ERK1/2 in the phosphorylation of MDR-1, A1847/MDR5 cells were labelled with $^{32}PO_4$ and then exposed to PD098059 or staurosporine, respectively, and the MDR-1 immunoprecipitated. No change in the level of phosphorylation of

 *© 2001 Cancer Research Campaign*

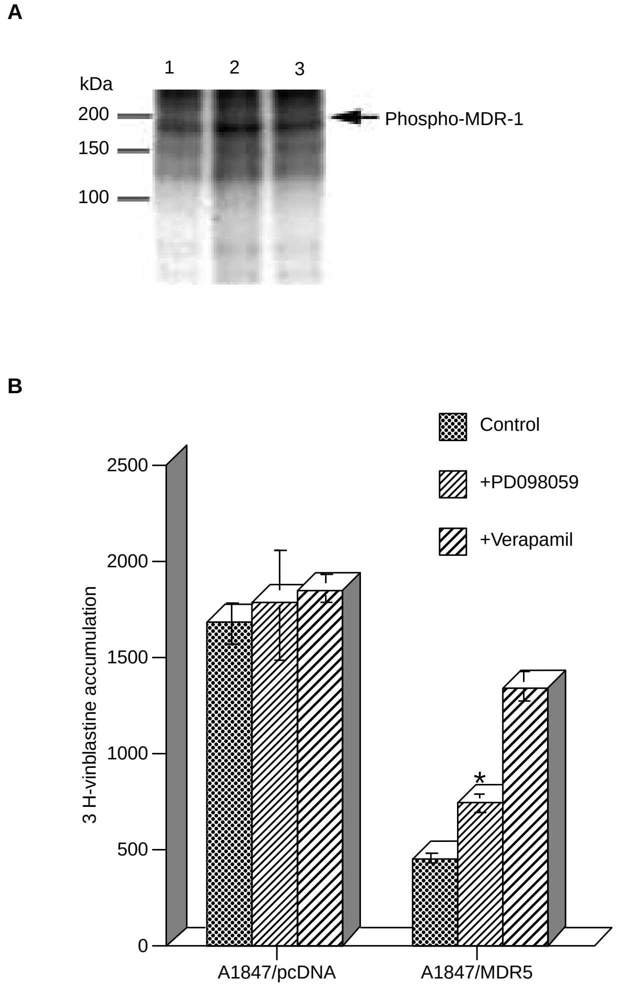

**Figure 5** Effect of ERK 1/2 MAP kinase inhibition on MDR-1 phosphorylation and function. (**A**) MDR-1 phosphorylation was determined by incubating A1847/MDR5 cells with labelled $^{32}PO_4$ alone (lane 1), in the presence of 30 $\mu$M PD098059 for 1 h (lane 2) or in the presence of 100 nM staurosporine for 1 h (lane 3). Phospho-MDR-1 was immunoprecipitated from the solubilized cells using the MDR-1 antibody C219. Immunoprecipitates (30 $\mu$g) were then analysed on 7% SDS-PAGE and the bands visualised by autoradiography. Further details are given in the Methods section. (**B**) Effect of ERK1/2 inhibition on drug accumulation. Control or A1847/MDR5 cells were incubated alone or in the presence of PD098059 (30 $\mu$M) or verapamil (25 $\mu$M) and the accumulation $^3$H-vinblastine was determined. Measurements were made 20 min after the substrate addition as described in Materials and Methods. The results shown are mean ± SD of at least 3 independent experiments. ** indicates statistical significance ($P < 0.05$) compared with the corresponding control

MDR-1 was observed (Figure 5A) indicating that activation of the ERK1/2 did not affect this aspect of MDR-1 function.

### *Transporter function*

Recently, it has been found that some inhibitors of PKC may block drug transport by direct competition with the transported drug rather than by effects on MDR-1 phosphorylation per se (Castro et al, 1999). We therefore studied the effects of PD098059 on drug transporter activity in the A1847/MDR5 cell line, using (G-$^3$H)-vinblastine as substrate. Radiolabelled Taxol was, unfortunately, unavailable for these experiments. In the wild-type cells, neither the MDR-1 reversing agent verapamil nor PD098059 had significant effect on drug accumulation which is consistent with the very low MDR-1 levels in these cells (Figure 5B). Drug accumulation was, however, lower in the A1847/MDR5 cell line using $^3$H-vinblastine as a substrate. Although verapamil caused a marked increase in drug accumulation, PD098059, which was equipotent in reversing the same degree of drug resistance, only increased accumulation of $^3$H-vinblastine by 60% of the appropriate control value (Figure 5B). Thus there is a poor correlation between the reversal of drug accumulation and the resulting increase in drug sensitivity. Nevertheless, PD098059 treatment may slightly increase the actual drug concentration in resistant cells and the reversal of Taxol resistance might partially result from this effect. In addition, the level of MDR-1 protein was unchanged upon treatment with PD098059 (not shown). Collectively, these data indicate that PD098059 is affecting some other function of the MDR-1, perhaps hitherto not ascribed to this protein or is acting by an alternative mechanism.

### DISCUSSION

In this paper we have investigated the ability of a range of signalling inhibitors to sensitise ovarian cancer cell lines to Taxol. Interestingly, all the inhibitors tested only reversed drug resistance significantly in cell lines overexpressing the drug transporter MDR-1. The effects were particularly marked in cell lines transfected with the Mdr-1 cDNA, indicating that the effects could be directly ascribed to the overexpression of this protein. The overexpression of MDR-1 resulted in the activation of the ERK 1 and ERK 2 pathways. This finding could rationalise the effects observed with the MAP kinase inhibitors PD098059 and U0126, as the activity of this pathway in the wild-type cell line was extremely low.

We could find little evidence that PD098059 could act as an MDR-1 antagonist, in the same manner that say verapmil does, therefore these effects in part could be ascribed to inhibition of the ERK1/2 pathway. However, there are a variety of mechanisms by which this could occur. In the first case, the ERK1/2 cascade is involved in the phosphorylation of a number of proteins, including transcription factors involved in determining cell survival and death (Hunter, 1997). PD098059 could therefore reverse changes in drug resistance mediated by this pathway directly. Alternatively, there may be direct cross-talk between the ERK1/2 signalling cascade and MDR-1 function i.e. that activation of the ERK1/2 either directly or indirectly affects the activity of the MDR-1 protein, by changing its phosphorylation state for example. However, we have been able to find no evidence of altered MDR-1 phosphorylation in cells grown in the presence of the PD098059 compound. In any event the role of phosphorylation of MDR-1 by other kinases, such as PKC, in modulating its function has also recently been called into question (Castro, 1999). It is feasible that the effects of PD098059 are by some alternative hitherto unknown mechanism. However, these would still be linked to Mdr-1 expression. The fact that PD098059 could reverse drug resistance to a greater extent than the alternative MEK inhibitor U0126 indicates that there may be an additional targets for this chemical. This is substantiated by recent evidence that PD098059 can interact with other cellular targets such as the Ah receptor and inhibit other signalling cascades such as those mediated by ERK 5 and the COX1/2 pathways for instance (Borsch-Haubold et al, 1998; Davies et al, 2000). In addition, Zuber et al (2000) report that in a genome-wide survey of Ras transformation targets, PD098059 treatment of cells has effects on 61 Ras targetted genes, blocking down-regulation of 36 genes and up-regulation of 25 targets

© 2001 Cancer Research Campaign

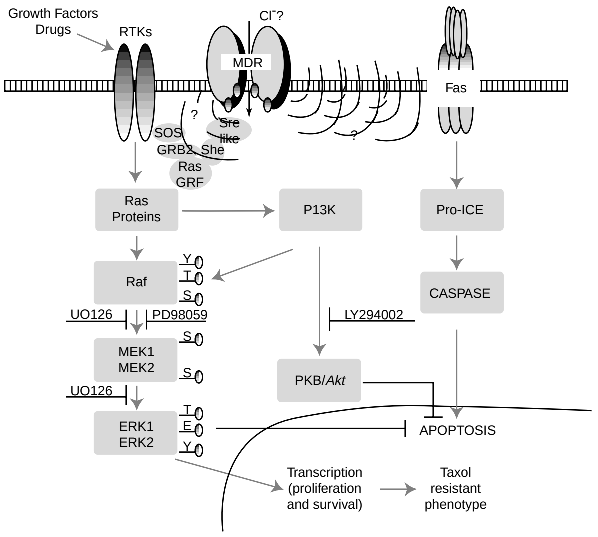

**Figure 6** Possible interactions between MDR-1 and ERK1/2 MAP kinase. Overexpression of MDR-1 in the cell membrane may result in several perturbations of intracellular signalling made evident through activation of the ERK1/2 MAP kinases including COX 1 and 2. Such changes in gene expression could have a profound effect upon the sensitivity of cell lines concomittantly exposed to chemotherapeutic agents. We suggest that the mechanism of action underlying certain of the changes observed in our own cell lines could be due, in part, to some of these 'extraneous' effects.

However, it is important to note that in all our studies to date the substantive increase in the drug sensitivity using ERK1/2 inhibitors has only been observed in cell lines overexpressing MDR-1. This indicates that the use of small molecule inhibitors, such as PD098059, to reverse drug resistance in cancer patients may be dependent on whether or not this transporter protein is overexpressed.

How MDR-1 results in the activation of ERK1/2 is unclear and may identify novel functions of this protein. Certain of these possible pathways are outlined in Figure 6. MDR-1, which is localised at the plasma membrane, may interact directly or indirectly with membrane-bound receptor tyrosine kinases (RTKs) known to be overexpressed in many tumours (Yao et al, 1988). Aberrant expression and/or activation of signal transducing proteins have been linked to various cancers. Over-expression of the epidermal growth factor receptor tyrosine kinase (c-erb-B1) gene has been observed in several human cancers including ovarian and breast (Yao et al, 1988). MDR-1 itself, particularly when over-expressed, might stimulate the interaction between these receptors and their effectors causing an activation or even inhibition of the downstream pathway. Indeed, it has been found that the expression of MDR-1 can block apoptosis induced by the Fas ligand cascade (Smyth et al, 1998). Expression of MDR-1 resulted in a decrease in the rate of production of active caspase-3, a key effector caspase in the apoptotic cascade, upon Fas ligation. Inhibition of MDR-1 function restored caspase 3 activation upon cross-linking of the cell surface Fas. However, cells retained their sensitivity to caspase-independent cell death stimuli regardless of their MDR-1 expression. It appears from these findings that the protection afforded by MDR-1 may in part be dependent upon the mechanism of action of the cell death mediator, such that it may protect from Taxol cytotoxicity for example but not that induced by other agents. The ERK1/2 pathway has also been shown to inhibit caspase-3 activation (Kim et al, 2000), so it is possible that some form of synergistic action results in enhanced drug resistance.

One further possibility is that MDR-1 may have additional physiological functions. MDR-1 induces novel $Na^+$ and $Cl^-$-dependent pathways for transmembrane $H^+$ efflux that results in intracellular alkalisation (Smyth et al, 1999). Apoptosis induced by chemotherapeutic agents is preceded by intracellular acidification and the induction of apoptotic events such as DNA laddering can be inhibited by increasing the intracellular pH in this manner. In a recent paper, Wittstein et al (2000) demonstrated that inhibition of the ERK 1/2 pathway using PD098059 resulted in re-alkalinisation of vascular endothelial cells in perfusion experiments. This effect was thought to be achieved through the effects of ERK upon anion exchange mechanisms at the cell surface. Thus alterations in cellular pH may directly or indirectly provide the stimulus for a variety of signalling pathways and responses mediated by both MDR and ERK1/2.

In addition to activation of the ERK1/2 pathway, activated Akt/PKB a possible mediator of the Taxol-resistant phenotype, was observed in the 1847/TX0.5 cell line made resistant to Taxol through media-conditioning. It is well known that PI3Ks play a central role in cell signalling cascades linked to cell survival. In mammalian cells there are several forms of PI3K with distinct functions and multiple downstream effectors including Src homology-2 (SH2), the PH domain of serine/threonine and tyrosine kinases and various cytoskeletal proteins (Franke et al, 1997). Inhibition of the PI3K, using the inhibitor of PI3K, LY294002, pathway resulted in a significant sensitisation to Taxol in the drug-resistant cells. However, no sensitisation was observed in the wild-type cells. This suggests that the increased PI3K activity could be linked to the overexpression of MDR-1 in the 1847/TX0.5 drug resistant cells. However, the activation of *Ak*t/PKB was not observed in the Mdr-1-transfected cell line, though resistance to Taxol was reversed using the LY294002 inhibitor. We concluded from this that PI3K/PKB activation is not a consequence of the MDR-1 overexpression but is linked to Taxol resistance by another mechanism.

Interestingly, Misra et al (1999) have demonstrated that PI3K-derived lipid products are actually necessary for maximal ATP-dependent transport across canalicular membrane vesicles. Inhibition of the pathway using wortmannin or LY294002 blocks this transport. The effect was not mediated by a direct effect upon the ATP transporter itself, but rather the inhibition of PI3K activity and changes mediated through the metabolism of lipid. It is conceivable that the effect observed in the 1847/TX0.5 and 1847/MDR5 cell lines is related to a perturbation in lipid metabolism.

Inhibition of either the PI3K or the p38 pathway alone had essentially no effect on wild-type cells. Inhibition of ERK1/2 using the PD098059 compound in wild-type cells resulted in only a 2-fold sensitisation to Taxol. Wild-type cells do not express detectable levels of MDR-1 and no activation of ERK1/2 is seen. The relative lack of effect is in contrast with reports that inhibition of these pathways has both a direct and potent anti-tumour action (Sebolt-Leopold et al, 1999; MacKeigan et al, 2000) and suggests that such effects may well be cell-type specific or ERK1/2 activation dependent. The generalised use of such inhibitors as single agents in chemotherapy must therefore be questionable. In all cell lines transfected with MDR-1, however, inhibition of the ERK1/2 MAP kinase and PI3K

 *© 2001 Cancer Research Campaign*

pathways by treatments combining Taxol with the appropriate inhibitors caused a marked (20–88-fold) increase in Taxol sensitivity. The effect was equivalent to the extensively used MDR-1 antagonist, verapamil. Furthermore, the combination of PD098059 with verapamil was synergistic causing a further 2-fold increase in sensitivity to this drug.

Collectively these data suggest that such signalling inhibitors may only be effective in chemotherapy when given in combination with other drugs and their effectiveness may well depend upon MDR-1 and ERK1/2 status.

### ACKNOWLEDGEMENTS

The authors would like to thank Dr Steve Keyse for critical reading of the manuscript.

The study was supported by Wellbeing & Royal College of Obstetricians & Gynaecologists Research Grant D1/95 and the Scottish Home and Health Department Grant Number K/MRS/50/C2626 for financial support.

### REFERENCES

Alessi DR, Cuenda A, Cohen P, Dudley DT and Saltiel AR (1995) PD098059 is a specific inhibitor of the activation of mitogen-activated protein kinase kinase in vitro and in vivo. *J Biol Chem* **270**: 27489–27494

Blagosklonny MV, Chuman Y, Bergan RC and Fojo T (1999) Mitogen-activated protein kinase pathway is dispensable for microtubule-active drug-induced Raf-1/Bcl-2 phosphorylation and apoptosis in leukemia cells. *Leukemia* **13**: 1028–1036

Bosch I and Groop J (1996) P-glycoprotein multidrug resistance and cancer. *Biochim Biophys Acta* **1288**: F37–F54

Castro AF, Horton JK, Vanoye CG and Altenberg GA (1999) Mechanism of inhibition of P-glycoprotein-mediated drug transport by protein kinase C blockers. *Biochem Pharmacol* **58**: 1723–1733

Davies SP, Reddy H, Caivano M and Cohen P (2000) Specificity and mechanism of action of some commonly used protein kinase inhibitors. *Biochem J* **351**: 95–105

Dent P, Jarvis WD, Birrer MJ, Fisher, PB, Schmidt-Ullrich RK and Grant S (1998) The roles of signaling by the p42/p44 mitogen-activated protein (MAP) kinase pathway; a potential route to radio-and chemo-sensitization of tumor cells resulting in the induction of apoptosis and loss of clonogenicity. *Leukemia* **12**: 1843–1850

Ding S, Yao D, Burchell B, Wolf CR and Friedberg T (1997) High levels of recombinant CYP3A4 expression in Chinese hamster ovary cells are modulated by coexpressed human P450 reductase and hemin supplementation. *Arch Biochem Biophys* **348**: 403–410

Emanuel SL, Chamberlin HA and Cohen D (1999) Antimitotic drugs cause increased tumorigenicity of multidrug resistant cells. *Inter J Oncology* **14**: 487–494

Eva A, Robbins KC, Andersen PR, Srinivasan A, Tronick SR, Reddy EP, Ellmore NW, Galen AT, Lautenberger JA, Papas TS, Westin EH, Wong-Staal F, Gallo RC and Aaronson SA (1982) Cellular genes analogous to retroviral onc genes are transcribed in human tumour cells. *Nature* **295**: 116–9119

Favata MF, Horiuchi KY, Manos EJ, Daulerio AJ, Stradley DA, Feeser WS, Van Dyk DE, Pitts WJ, Earl RA, Hobbs F, Copeland RA, Magolda RL, Scherle PA and Trzaskos JM (1998) Identification of a novel inhibitor of mitogen-activated protein kinase kinase. *J Biol Chem* **273**: 18623–18632

Franke TF, Kaplan DR and Cantley LC (1997) P13K: downstream AKTion blocks apoptosis. *Cell* **88**: 435–437

Hayakawa J, Ohmichi M, Kurachi H, Ikegami H, Kimura A, Matsuoka T, Jikihara H, Mercola D and Murata Y (1999) Inhibition of extracellular signal-regulated protein kinase or c-Jun N-terminal protein kinase cascade, differentially activated by cisplatin, sensitizes human ovarian cancer cell line. *J Biol Chem* **274**: 31648–31654

Hoshino R, Chatani Y, Yamori T, Tsuruo T, Oka H, Yoshida O, Shimada Y, Ari-i S, Wada H, Fujimoto J and Kohno M (1999) Constitutive activation of the 41/43-kDa mitogen-activated protein kinase signaling pathway in human tumors. *Oncogene* **18**: 813–822

Johnstone RW, Cretney E and Smyth MJ (1999) P-Glycoprotein protects leukaemia cells against caspase-dependent, but not caspase-independen, cell death. *Blood* **93**: 1075–1085

Kauffmann-Zeh A, Rodriguez-Viciana P, Ulrich E, Gilbert C, Coffer P, Downward J and Evan G (1997) Suppression of c-Myc-induced apoptosis by Ras signalling through PI(3)K and PKB. *Nature* **385**: 544–548

Keyse SM (1995) An emerging family of dual specificity MAP kinase phosphatases. *Biochim Biophys Acta* **1265**: 152–160

Kim M, So H, Park J, Lee K, Moon B, Lee H, Kim T, Moon S and Park R (2000) Hwansodan protects PC12 cells against serum-deprivation-induced apoptosis via a mechanism involving Ras and mitogen-activated protein (MAP) kinase pathway. *Gen. Pharmacol* **34**: 227–235

Kinloch RA, Treherne JM, Furness LM and Hajimohamadreza I (1999) The pharmacology of apoptosis. *Trends Pharmacol Sci* **20**: 35–42

Kioka N, Tsubota J, Kakehi Y, Komano, T, Gottesman MM, Pastan I and Ueda K (1989) P-glycoprotein gene (MDR-1) cDNA from human adrenal: normal P-glycoprotein carries Gly185 with an altered pattern of multidrug resistance. *Biochem Biophys Res Comm* **162**: 224–231

Leevers SJ, Vanhaesebroeck B and Waterfield MD (1999) Signalling through phosphoinositide 3-kinases: the lipids take centre stage. *Curr Opin Cell Biol* **11**: 219–225

Ling Y-H, Yang Y, Tornos C, Singh B and Perez-Soler R (1998) Paclitaxel-induced apoptosis is associated with expression and activation of c-Mos gene product in human ovarian carcinoma SKOV3 cells. *Cancer Res* **58**: 3633–3640

MacKeigan JP, Collins TS and Ting JP-Y (2000) MEK inhibition enhances paclitaxel-induced tumor apoptosis. *J Biol Chem* **275**: 38953–38956

Mansour SJ, Matten WT, Hermann AS, Candis JM, Rong S, Fukasawa K, Vande Woude GF and Ahn AG (1994) Transformation of mammalian cells by constitutively active MAP kinase kinase. *Science* **265**: 966–970

Misra S, Ujhazy P, Varticovski L and Arias IM (1999) Phosphoinostide 3-kinase lipid products regulate ATP-dependent transporter by sister of P-glycoprotein and multidrug resistance associated protein 2 in bile canalicular membrane vesicles. *Proc Natl Acad Sci USA* **96**: 5814–5819

Mosmann T (1983) Rapid colorimetric assay for cellular growth and survival: application to proliferation and cytotoxicity assays. *J Immuno Methods* **65**: 55–63

Parekh H, Wiesen K and Simpkins H (1997) Acquisition of Taxol resistance via P-glycoprotein- and non-P-glycoprotein-mediated mechanisms in human ovarian carcinoma cells. *Biochem Pharmacol* **53**: 461–470

Plo I, Bettaieb A, Payrastre B, Mansat-De Mas V, Bordier C, Rousse A, Kowalski-Chauvel A, Laurent G and Lautier D (1999) The phosphoinositide 3-kinase/Akt pathway is activated by daunorubicin in human acute myeloid leukemia cell lines. *FEBS Letters* **452**: 150–154

Salh BS, Martens J, Hundal RS, Yoganathan N, Charest D, Mui A and Gomez-Munoz A (2000) PD98059 attenuates hydrogen peroxide-induced cell death through inhibition of Jun N-terminal Kinase in HT29 cells. *Mol Cell Biol Res Commun* **3**: 158–165

Sandor V, Fojo T and Bates SE (1998) Future perspectives for the development of P-glycoprotein modulators. *Drug Resistance Updates* **1**: 190–200

Sarris AH, Younes A, McLaughlin P, Moore D, Hagemeister F, Swan F, Rodriguez MA, Romaguera J, North L, Mansfield P, Callendar D, Mesina O and Cabanillas F (1996) Cyclosporin A does not reverse clinical resistance to paclitaxel in patients with relapsed non-Hodgkin's lymphoma. *J Clin Oncol* **14**: 233–239

Scheid MP and Woodgett JR (2000) Protein kinases: six degrees of separation. *Curr Biol* **10**: R191–R194

Schulte TW, Blagosklonny MV, Romanova L, Mushinski JF, Monia BP, Johnston JF, Nguyen P, Trepel J and Neckers LM (1996) Destabilization of Raf-1 by geldanamycin leads to disruption of the Raf-1-MEK-mitogen-activated protein kinase signalling pathway. *Mol Cell Biol* **16**: 5839–5845

Sebolt-Leopold JS, Dudley DT, Herrera R, Becelaere KV, Wiland, A, Goman RC, Tecle H, Barrett SD, Bridges A, Przybranowski S, Leopold WR and Saltiel AR (1999) Blockade of the MAP kinase pathway supresses growth of colon tumors *in vivo*. *Nature Med* **5**: 810–816

Shayesteh L, Lu Y, Kuo WL, Baldocchi R, Godfrey T, Collins C, Pinkel D, Powell B, Mills GB and Gray JW (1999) PIK3CA is implicated as an oncogene in ovarian cancer. *Nature Genetics* **21**: 99–102

Smith CD and Zilfou JT (1995) Circumvention of P-glycoprotein-mediated multiple drug resistance by phosphorylation modulators is independent of protein kinases. *J Biol Chem* **270**: 28145–28152

Smyth MJ, Krasovskis E, Sutton VR and Johnstone RW (1998) The drug efflux protein, P-glycoprotein, additionally protects drug-resistant tumor cells from multiple forms of caspase-dependent apoptosis. *Proc Natl Acad Sci USA* **95**: 7024–7029

*© 2001 Cancer Research Campaign* 

Tsuruo T, Iida H, Tsukagoshi S and Sakurai Y (1981) Overcoming of vincristine resistance in P388 leukemia in vivo and in vitro through enhanced cytotoxicity of vincristine and vinblastine by verapamil. *Cancer Res* **41**: 1967–1972

Wang X, Martindale JL, Liu Y and Holbrook NJ (1998) The cellular response to oxidative stress: influences of mitogen-activated protein kinase signalling pathways on cell survival. *Biochem J* **333**: 291–300

Wennstrom S and Downward J (1999) Role of phosphoinositide 3-kinase in activation of ras and mitogen-activated protein kinase by epidermal growth factor. *Mol Cell Biol* **19**: 4279–4288

Wittstein IS, Qiu W, Ziegelstein RC, Hu Q and Kass DA (2000) Opposite effects of pressurized steady versus pulsatile perfusion on vascular endothelial cell cytosolic pH: role of tyrosine kinase and mitogen-activated protein kinase signaling. *Circ. Res* **86**: 1230–1236

Yao M, Shuin T, Misaki H and Kubota Y (1988) Enhanced expression of c-myc and epidermal growth factor receptor (c-erbB-1) genes in primary human renal cancer. *Cancer Res* **48**: 6753–6757

Yuan ZQ, Sun M, Feldman RI, Wang G, Ma X, Jiang C, Coppola D, Nicosia SV and Cheng JQ (2000) Frequent activation of AKT2 and induction of apoptosis by inhibition of phosphoinositide-3-OH kinase/Akt pathway in human ovarian cancer. *Oncogene* **19**: 2324–2330

Zuber J, Tchernitsa OI, Hinzmann B, Schmitz AC, Grips M, Hellriegel M, Sers C, Rosenthal A and Schafer R (2000) A genome-wide survey of RAS transformation targets. *Nat Genet* **24**: 144–152

*© 2001 Cancer Research Campaign*
